# Rapid Classification of Multilocus Sequence Subtype for Group B *Streptococcus* Based on MALDI-TOF Mass Spectrometry and Statistical Models

**DOI:** 10.3389/fcimb.2020.577031

**Published:** 2021-01-29

**Authors:** Lianfen Huang, Kankan Gao, Guanglian Chen, Huamin Zhong, Zixian Li, Xiaoshan Guan, Qiulian Deng, Yongqiang Xie, Wenjing Ji, David J. McIver, Chien-Yi Chang, Haiying Liu

**Affiliations:** ^1^ Clinical Laboratory, Guangzhou Women and Children’s Medical Center, Guangzhou Medical University, Guangzhou, China; ^2^ Department of Pharmacy Administration and Clinical Pharmacy, School of Pharmacy, Xi’an Jiaotong University, Xi’an, China; ^3^ Global Health Group, Institute for Global Health Sciences, University of California, San Francisco, San Francisco, CA, United States; ^4^ School of Dental Sciences, Newcastle University, Newcastle upon Tyne, United Kingdom

**Keywords:** group B Streptococcus, MALDI-TOF/MS, statistical model, multi-locus sequence typing, sequence type

## Abstract

Group B *Streptococcus* (GBS) is an important etiological agent of maternal and neonatal infections as well as postpartum women and individuals with impaired immunity. We developed and evaluated a rapid classification method for sequence types (STs) of GBS based on statistic models with Matrix-Assisted Laser Desorption/Ionization Time-of Flight Mass Spectrometry (MALDI-TOF/MS). Whole-cell lysates MALDI-TOF/MS analysis was performed on 235 well-characterized GBS isolates from neonatal invasive infections in a multi-center study in China between 2015 and 2017. Mass spectra belonging to major STs (ST10, ST12, ST17, ST19, ST23) were selected for model generation and validation. Recognition and cross validation values were calculated by Genetic Algorithm-K Nearest Neighbor (GA-KNN), Supervised Neural Network (SNN), QuickClassifier (QC) to select models with the best performance for validation of diagnostic efficiency. Informative peaks were further screened through peak statistical analysis, ST subtyping MSP peak data and mass spectrum visualization. For major STs, the ML models generated by GA-KNN algorithms attained highest cross validation values in comparison to SNN and QC algorithms. GA-KNN models of ST10, ST17, and ST12/ST19 had good diagnostic efficiency, with high sensitivity (95–100%), specificity (91.46%–99.23%), accuracy (92.79–99.29%), positive prediction value (PPV, 80%–92.68%), negative prediction value (NPV, 94.32%–99.23%). Peak markers were firstly identified for ST10 (m/z 6250, 3125, 6891) and ST17 strains (m/z 2956, 5912, 7735, 5218). Statistical models for rapid GBS ST subtyping using MALDI-TOF/MS spectrometry contributes to easier epidemical molecular monitoring of GBS infection diseases.

## Introduction

Group B *Streptococcus* (GBS), a gram-positive coccus commonly colonizing in the female lower genital tract or rectum, is one of the most common causes of sepsis, meningitis and pneumonia in neonates and fetal injury, preterm birth, spontaneous abortion in pregnant women (Lamagni et al., 2013; Nanduri et al., 2019), or invasive infections in postpartum women (endometritis) and individuals with impaired immune systems (Tevdorashvili et al., 2015), leaving poor prognosis such as neonatal death, severe neutral system sequelae, etc. GBS colonization in pregnant women is a major risk factor for neonatal and infant infections (Burcham et al., 2019). The most common epidemiological analysis of GBS infections is the latex agglutination (LA) assay using the specific surface capsular polysaccharide (CPS) antibodies for serologic typing (El et al., 2019). The commercial LA kits sometimes are unable to serotype the GBS isolates with low expression of CPS, methods for geno-serotyping with both serotyping and PCR amplification of the capsular gene was developed accordingly (Poyart et al., 2007; Imperi et al., 2010). However, the high expense of geno-serotyping makes it difficult for routine clinical analysis. Moreover, GBS strains belonging to identical capsular serotype III or Ib have shown significant differential characteristics in phenotype and genotype (Arias et al., 2019; Wu et al., 2019; Zheng et al., 2020). Ribotyping is not widely applied for determination of rDNA RFLP patterns, it can be used as a typing system only in conjunction with serotyping (Huet et al., 1993), and this methods is not always comparable among different laboratories.

Multilocus sequence typing (MLST) has been widely used as another powerful tool to study the genetic lineages of GBS strains based on the gene sequence of seven housekeeping genes since 2003 (Jones et al., 2003). By using MLST, the majority sequence types (STs) of the human GBS isolates were ST1, ST10, ST12, ST17, ST19, ST23, and ST26 (Da et al., 2014; Wu et al., 2019; Li et al., 2019; McGee et al., 2020). Special GBS STs were found to be strongly associated with neonatal invasive diseases (Wu et al., 2019; Li et al., 2019). For example, the ST17 clone, mostly serotype III, expressing hypervirulent adhesin (HvgA) as a signature virulence factor for this lineage, has a greater potential to cause invasive diseases like neonatal sepsis and meningitis, accounting for more than 80% of late-onset (LOD) meningitis cases (Manning et al., 2009; Landwehr-Kenzel and Henneke, 2014; Zhu et al., 2020). High prevalence of hypervirulent clone ST17 among pregnant and non-pregnant patients could be sources of further neonatal infections (Kardos et al., 2019). While other STs like ST1, ST23, and ST19, the predominant colonizers in pregnant women, are well adapted to vaginal mucosa with a limited invasive potential in neonates (Teatero et al., 2017). Besides, different STs have differential drug resistant characteristics. ST17 tended to be levofloxacin-susceptible but *tet*O-positive resulting in tetracycline resistance, and ST19 tended to be levofloxacin-resistant but *tet*O-negative (Wang X. et al., 2018), while ST10 showed high radezolid MICs (Zheng et al., 2020) and FQ resistance (Arias et al., 2019). Thus, it is meaningful for rapid ST typing of GBS, which could facilitate an optimal clinical treatment such as antibiotic selection guidance for neonates and pregnant women with GBS infection or colonization. As the traditional MLST analysis is laborious, time-consuming and costly, it is necessary to develop more friendly, less expensive and faster GBS typing methods in clinical microbiology laboratories.

Matrix-assisted laser desorption ionization-time of flight mass spectrometry (MALDI-TOF/MS), a transformative proteomics for clinical microbiology (Patel, 2013), has been widely used for a rapid, low-cost, and accurate microbial species identification based on their unique protein profiles (Bizzini and Greub, 2010; Ohkusu, 2012; Nomura, 2015). The application of this technology is successfully broaden from species identification to subtyping within pathogen species, including *Escherichia coli*, *Staphylococcus aureus*, *Streptococcus pneumoniae*, *S. pyogenes* (Moura et al., 2008; Williamson et al., 2008; Wang et al., 2012; Nakano et al., 2015; Sauget et al., 2017). For GBS, several MALDI-TOF/MS peak biomarkers have been identified for rapid recognition of ST1 and ST17 clones (Lartigue et al., 2011; Lanotte et al., 2013; Lin et al., 2019). Recent study also has developed a GBS subtyping based on mass variation of ribosomal subunit proteins (rsp profile) by MALDI-TOF/MS (Rothen et al., 2019), but it is far from routine clinical application. Moreover, rapid classification of GBS serotypes based on MALDI-TOF MS spectrometry and machine learning techniques could attain 55% to 87% accuracies for the five main serotypes (Ia, Ib, III, V, VI) (Wang et al., 2019), their predictive efficiency was not powerful enough for clinical application except for serotype III (according to our external validation data which was not shown in this study). In this study, with hundreds of GBS mass spectra belonging to various STs, statistical classification models were generated and validated to evaluate their predictive abilities on GBS STs by MOLDI-TOF/MS. The main informative peaks for the discrimination of major STs were also analyzed.

## Materials and Methods

### Bacterial Isolates

This study involved 235 clinical GBS isolates from infants (≤90 days) with invasive infections in China from 2015 to 2017, as reported at the previous multi-center study (Ji et al., 2019). The isolates were representative of the main epidemic ST clones in China. All isolates were recovered from blood or cerebrospinal fluid (CSF) cultures after routine identification in clinical microbiology laboratories of tertiary healthcare centers, and were characterized by MLST and serotyping (Ji et al., 2019; Li et al., 2019) (**Table S1**). Pre-analysis of the total dataset identified 31 types of STs, the most prevalent STs were ST10, ST12, ST17, ST19, ST23, the major serotypes were III, Ib, and Ia, as well as a strong association of serotype III with ST17/ST19, serotype Ib with ST10/ST12 and serotype Ia with ST23. Sequence type allelic profile of seven housekeeping genes (*pheS*, *atr*, *tkt*, *glcK*, *sdhA*, *glnA*, *adhP*) of the 235 GBS isolates was generated by STRAT2 software (https://pubmlst.org/software/analysis/start2/) (Jolley et al., 2001) (**Figure S1**), which was based on the unweighted-pair group method using average (UPGMA) linkages and represented as a dendritic tree showing the genetic relationships among 31 ST subtypes. This study was approved by the Ethics Committee of Guangzhou Women and Children’s Medical Center (2017021915) for analyses of clinical isolates.

### Sample Preparation and MALDI-TOF/MS Data Acquisition

Bacterial cultures were grown for 16 to 18 h on Columbia sheep blood agar at 37°C in an incubator with 5% CO_2_. The batch effect on MALDI-TOF measurement has raised considerable concern in applying machine learning (ML) for MALDI-TOF spectra analysis. Our pretest suggested that large quantities of peaks, especially the ones with low intensities that were probably associated with phylogenetic lineages (Lartigue et al., 2009), could only be easily detected and reproduced through in-tube ethanol/formic acid extracted protein microorganism mass spectrum profiling (Lartigue et al., 2009; Lartigue et al., 2011) in comparison with the mass spectra acquired by direct bacteria deposit, the most frequent way for routine bacteria identification (Wang H. Y. et al., 2018). Therefore, we applied the ethanol/formic acid in-tube extracted microorganism profiling after standard procedures for bacterial culture, using the same settings for mass spectra collection and preparation, as well as selecting mass spectra with excellent logarithm score [*log(s)*≥2.3], etc, to lower the possible batch effect that may occur when applying ML for MALDI-TOF spectra analysis. All GBS isolates were subjected to microorganism profiling ethanol/formic acid extraction as previously described (Lartigue et al., 2009). MALDI-TOF/MS was performed on a MALDI Microflex LT (Bruker Daltonics, Bremen, Germany) instrument running FlexControl 3.0 software. External calibration was performed with Bruker Bacterial Test Standard. For MALDI-TOF/MS analysis, 1µl aliquot bacterial protein extract for each strain was spotted onto a MALDI target plate and air-dried at room temperature, 2 µl aliquot of the α-cyano-4-hydroxycinnamic acid (HCCA) matrix solution [saturated, 50% acetonitrile (CAN)-2.5 trifluoroacetic acid (TFA)] (Barbuddhe et al., 2008) was overlaid on every sample spot of the MALDI-target plate and air-dried for analysis within 30 minutes. Experiments from cultures to protein extraction was repeated twice. The ion spectra of peptide mass fingerprints products were recorded for three times for every isolate by MALDI-TOF MS in positive linear mode. For each spectrum, 240 laser shots were automatically collected from different positions per sampling area of the target spot at 60 Hz in 40 shots (random walk movement).

The data of the raw spectra were aligned to mass spectrum profile (MSP) of GBS strains in MALDI Biotyper reference database (*S.agalactiae* CTL03_102-ST1, *S.agalactiae* CTL03_145-ST17, *S.agalactiae* CTL03_198-ST19, *S.agalactiae* CTL04_158-ST8, *S.agalactiae* CTL CNR10-ST23, DSM 16828, DSM2134T, DSM6784, CTL V29-ST10) using MALDI Biotyper 2.1 software (Bruker Daltonik GmhH, Bremen, Germany) through the integrated pattern-matching algorithm of the software (Lartigue et al., 2009). The detected prominent ion peaks list included 70 peaks between 2,000 and 20,000 Da. As previous report (Lartigue et al., 2009), the logarithm score [*log(S)*] of the MALDI Biotyper pattern-matching algorithm was calculated (MALDI Biotyper 2.1 software), all 235 isolates were validated to be GBS at the species level [*log(S)*≥2.0]. To increase the model performance and simplify the subtyping procedure, a model validation pretest for four GBS isolates assigned to ST10, ST12, ST17, ST19 respectively, each with 20 replicates of qualified mass spectrum [*log(S)*≥2.0], showed mass spectra with excellent scores [*log(S)*≥2.3] (Lartigue et al., 2009) displaced better ST subtyping performance both in reproducibility and accuracy. Therefore, mass spectra with excellent scores [*log(S)*≥2.3] were selected for all isolates for subsequent model generation, validation, and ST subtyping peak analysis by statistic and MSP.

### MALDI-TOF/MS Data Analysis

The raw GBS spectra were firstly modified by both spectra smoothing and baseline subtraction on FlexAnalysis 3.0 software for one time before their loading on CliniProTools for peak statistics, model generation, validation and classification evaluation, as well as MALDI BioTyper for MSP peak description. Then, the modified MALDI-TOF MS spectra of all isolates were loaded to ClinProTools software and grouped into different ST classes (ST10, ST12, ST17, ST19, ST23, other STs) to produce their gel and peak statistics views. Statistical classification models were generated and functioned as classifiers, which could classify the new spectra with unknown ST types. Spectra preparations on CliniProTools were set as default setting in the manual user (Bruker Daltonik GmbH, 2011) after their loading including spectra smoothing, baseline subtraction, recalibration, null spectra exclusion in applying ML for analyzing MALDI-TOF spectra (Wang H. Y. et al., 2018). Null spectra were replaced with valid spectra. One mass spectrum [*log(S)*≥2.3] of all GBS strains belonging to four main STs (ST10, ST12, ST17, ST19) were applied for peak statistical analysis. Values of m/z from their average spectra were extracted, significant differential peaks were identified according to their statistical significance through three statistical tests on ClinProTools including Anderson–Darling (AD) test, t-/Analysis of Variance (ANOVA) test (PTTA), Wilcoxon/Kruskal–Wallis tests (PWKW). Characteristic peaks were selected for a normally distributed data (p-value AD >0.05, PTTA or PWKW ≤0.05) and non-normally distributed data (p-value AD ≤ 0.05 and PWKW ≤0.05) (Stephens, 1974; Camoez et al., 2016; Wang H. Y. et al., 2018).

Before model generation, 235 GBS mass spectra from every 235 GBS isolates were divided into six ST classes (ST10, ST12, ST17, ST19, ST23, and other STs), then they were loaded to get a 2D peak statistic distribution overview enabled by their top two differential peaks (Bruker Daltonik GmbH user manual, 2011) for visualization of mass spectra peak profile characteristics among different STs. Meanwhile, the same six classes of mass spectra were used for MSP peak analysis with MALDI BioTyper 2.1 to acquire their peak data with average intensity and frequency. For all 235 GBS mass spectra, their major STs (ST10, ST12, ST17, ST19, ST23) were randomly divided into a training set and an external validation set for all ST models. Mass spectra in both datasets were furtherly assigned into two classes with different ST subtypes for model generation and external validation (**Table 1**). As for classification problems considered with CliniProTools, the analysis must be applied on a set of independent spectra where intervention and no intervention are not mixed, only one mass spectra were applied for every GBS strains in both training and validation groups. For ML models of different STs, a 2D peak statistic distribution overview was produced when two corresponding ST classes of mass spectra were loaded as the training set for model calculations on ClinProTools 3.0. Classification models were generated and optimized for performance evaluation through three open algorithms in CliniProTools, including Genetic Algorithm-K Nearest Neighbor (GA-KNN), Supervised Neural Network (SNN), or QuickClassifier (QC) (Bruker Daltonik GmbH, 2011), the optimization details for GA-KNN algorithm was shown in the supplemental material (**Table S2**). All peaks in the spectra were picked in model generation, the W/KW test was used for peak selection. The special setting methods of the ML models were described in detail in the ClinProTools 3.0 user manual (Bruker Daltonik GmbH, 2011). Random mode was chosen for calculating cross validation of generated ML models (Bruker Daltonik GmbH user manual, 2011). The recognition capability and cross validation values were calculated to access the performance of ML models. The optimized ML models with good performances were selected as the final models for further external validation and classification evaluation. The validation datasets with known STs were loaded to evaluate the diagnostic efficiency of the optimal models in cross-validation evaluation. Five indexes were calculated to evaluate the performance of the predictive models, including sensitivity (Sn), specificity (Sp), accuracy (Acc), positive prediction value (PPV), and negative prediction value (NPV).

**Table 1:** Data grouping for ML analysis of GBS ST subtypes based on MALDI-TOF spectra using GA-KNN algorithm.

Train	Group 1 (n = no. of isolates)		Group 2 (n = no. of isolates)		Additional data set
	Training	Validation	Training	Validation	Classification
ST10	ST10 (n = 20)	ST10 (n = 11)	ST12 (n = 10)	ST12 (n = 22)	Other STs (n = 39)
			ST17 (n = 10)	ST17 (n = 75)	
			ST19 (n = 10)	ST19 (n = 27)	
			ST23 (n = 5)	ST23 (n = 6)	
ST12	ST12 (n = 20)	ST12 (n = 12)	ST10 (n = 10)	ST10 (n = 21)	ST23 (n = 11)
			ST17 (n = 10)	ST17 (n = 75)	Other STs (n = 39)
			ST19 (n = 10)	ST19 (n = 27)	
ST17	ST17 (n = 45)	ST17 (n = 40)	ST12 (n = 15)	ST12 (n = 16)	ST23 (n = 11)
			ST17 (n = 15)	ST17 (n = 17)	Other STs (n = 39)
			ST19 (n = 15)	ST19 (n = 22)	
ST19	ST19 (n = 20)	ST17 (n = 17)	ST10 (n = 10)	ST10 (n = 21)	ST23 (n = 11)
			ST12 (n = 10)	ST12 (n = 22)	Other STs (n = 39)
			ST17 (n = 10)	ST17 (n = 75)	
ST12/ ST19	ST12 (n = 20)	ST12 (n = 12)	ST10 (n = 20)	ST10 (n = 11)	Other STs (n = 39)
	ST19 (n = 20)	ST19 (n = 17)	ST17 (n = 20)	ST17 (n = 65)	
			ST23 (n = 5)	ST23 (n = 6)	

Other STs (n = 39): ST2 (n = 2), ST4, ST8, ST24 (n = 2), ST27 (n = 6), ST55, ST88, ST103, ST138, ST146, ST156, ST163, ST179, ST188 (n = 5), ST197 (n = 2), ST223, ST249, ST268, ST335, ST357, SST452, ST480, ST579, ST651 (n = 2), ST680, ST938.

Informative peaks from all ML models were described in combination with peak statistic data of the four major STs (ST10, ST12, ST17, ST19) on ClinProTools, as well as MSP ST peak data with average intensity and frequency for all six ST classes (ST10, ST12, ST17, ST19, ST23, other STs) for identification of potential MALDI-TOF/MS peak biomarkers for certain STs, which were furtherly evaluated by spectra visualization on FlexAnalysis 3.0 software. To examine the performance of the optimal ML models when challenged against isolates belonging to STs that were not included in the model, an additional set with three minor STs (ST23, ST27, ST188) and 24 sporadic STs (ST2, ST4, ST8, ST24, ST55, ST88, ST103, ST138, ST146, ST156, ST163, ST179, ST197,ST223, ST249, ST268, ST335, ST357, SST452, ST480, ST579, ST651, ST680, ST938) were selected and loaded as a classification set on CliniProTools for calculation and classification evaluation through the same algorithm (GA-KNN). Duplicate mass spectra collected from every GBS strains in the classification set were loaded for classification to roughly evaluate the reproducibility of the generated ML models on classifying these minor STs and sporadic STs.

## Results

### Gel/Stack and Peak Statistical Overview of the Total Data Sets

**Figure 1:**
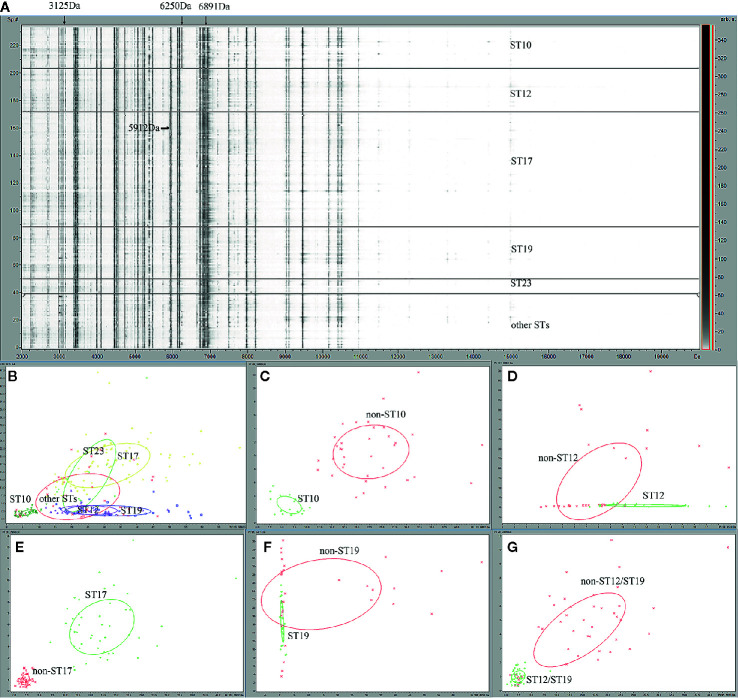
Gel overview and peak statistic of the GBS MALDI-TOF mass spectra for different ST classes of GBS strains. **(A)** Gel overview **(B)** Peak statistic overview of the 235 mass spectra for six ST classes of GBS strains. ST10 (dark green), ST12 (purple), ST17 (yellow), ST19 (blue), ST23 (light green), other STs (red). **(C)** Peak statistic overview of the spectra for ST10 model, ST10 (green), non-ST10(red); **(D)** ST12 model, ST12 (green), non-ST12 (red); **(E)** ST17 model, ST17 (green), non-ST17(red); **(F)** ST19 models, ST19 (green), non-ST19 (red); **(G) **ST12/ST19 model, ST12/ST19 (green), non-ST12/ST19 (red). The other STs group included 39 GBS isolates belong to 26 other ST types ST2, ST4, ST8, ST24, ST27 , ST55, ST88, ST103, ST138, ST146, ST156, ST163, ST179, ST188, ST197 , ST223, ST249, ST268, ST335, ST357, SST452, ST480, ST579, ST651, ST680, ST938.

For the 235 GBS isolates, a gel/stack view of whole cell lysate protein profiles (**Figure 1A**) was generated from the pre-processed mass spectra for characteristic overview of the six ST classes (ST10, ST12, ST17, ST19, ST23, and other STs). Group of other STs was composed of 26 ST minor subtypes (**Table 1**). From the gel/stack view, there were clear similarities and differences among the mass spectra of different STs. For differentiation, presence of m/z 3125, and m/z 6250 and absence of m/z 6891 were specific to ST10 strains (**Figure 1A**). A 2D peak statistic overview of the six loaded ST classes was shown enabled by the top two differential peaks among them (**Figure 1B**), indicating a good separation among ST10, ST17, ST12/ST19 classes. Clear separation of binary classes was observed by 2D peak statistic view of the training data for ST10 ML models (**Figure 1C**), ST17 (**Figure 1E**), and ST12/ST19 (**Figure 1G**). Noticeably, for 2D peak statistic overview of mass spectra in ST12/ST19 model enabled by their top two differential peaks m/z 2956 and 5912, some of the plots (belonging to ST10) of class 2 (ST10, ST17, and ST23), seemed to mix together with the plots of class 1 (ST12 and ST19) (**Figure 1G**). These plots (ST10) could be easily separated with class 1 (ST12 and ST19) just through the third most powerful differential peak m/z 6250. As for the partial peak overlapping between ST23 and ST12/ST17/ST19, as well as between other STs group (containing 26 minor STs) and the five main STs (ST10, ST12, ST17, ST19, ST23), ST23, and other STs were not included in the training set for generation of ST12, ST17, and ST19 models. Due to the great overlapping of 2D peak statistical view between ST12 and ST19, we evaluated a ST12/ST19 GA model that took ST12 and ST19 as one group in order to correctly identify ST12 and ST19 subclones before their further classification by ST12 and ST19 models.

### Peak Statistic and Cross-Validation of the Predictive Models

Peak statistical analysis identified 29 statistically differential peaks among MS spectra data of the four main STs (ST10, ST12, ST17, ST19) used for model generation (see supplemental xls1). Five predictive models were generated, including ST10 versus non-ST10 (**Figure 1C**), ST12 vs non-ST12 (**Figure 1D**), ST17 vs non-ST17 (**Figure 1E**), ST19 vs non-ST19 (**Figure 1F**), ST12/ST19 vs non-ST12/ST19 (**Figure 1G**). Spectra grouping for model generation was provided in **Table 1**.

In this study, the performance of the predictive models for major STs (ST10, ST12, ST17, ST19) by GA-KNN, SNN and QC algorithms were shown in **Table S2**. The predictive models generated by GA algorithms (GA-KNN) attained both higher recognition and cross validation values than using SNN and QC algorithms, indicating a better reliability and predictive ability of the calculated models by GA-KNN algorithms. Besides, optimization of best differential peak combinations maximum number (10,20,30) and KNN numbers (1, 3, 5, 7) for GA-KNN algorithm during model generation showed nearly all ST GA-SNN models could attain optimal performances using 10 instead of 20 and 30 as the maximum number of best differential peak combinations and 3 as KNN numbers for all major ST lineages (**Table S2**). This was in accordance with the description in user manual that a reasonable reduction of peaks always could improve the classification performed by the algorithm (Bruker Daltonik GmbH, 2011). In this study, for easier model optimization and seeking more differential peaks, five optimized machine-learning (ML) models calculated by GA10-KNN3 algorithm were selected as the final optimized pattern recognition models for the following external validation and classification evaluation, including ST10-GA(10)-KNN3 for ST10, ST12-GA(10)-KNN3 for ST12, ST17-GA(10)-KNN3 for ST17, ST19-GA(10)-KNN3 for ST19, ST12/ST19-GA(10)-KNN3 for ST12/ST19 (**Table S2**), which were referred as ML models using GA-KNN algorithm for ST10, ST12, ST17, ST19, ST12/ST19 afterwards. Cross validation demonstrated that ST10 GA-KNN and ST17 GA-KNN with cross validation value 98.72% and 98.37%, respectively, were reliable and had best predictive abilities, while ST12/ST19 GA-KNN model that took ST12 and ST19 as one group with cross validation value 97.92% could help correctly recognize mass spectra of ST12 and ST19 subclones before their further classification by ST12 GA-KNN and ST19 GA-KNN models with cross validation value 81.19% and 75.42% respectively (**Table S2**).

### Informative Peaks in Each Predictive Model

**Figure 2:**
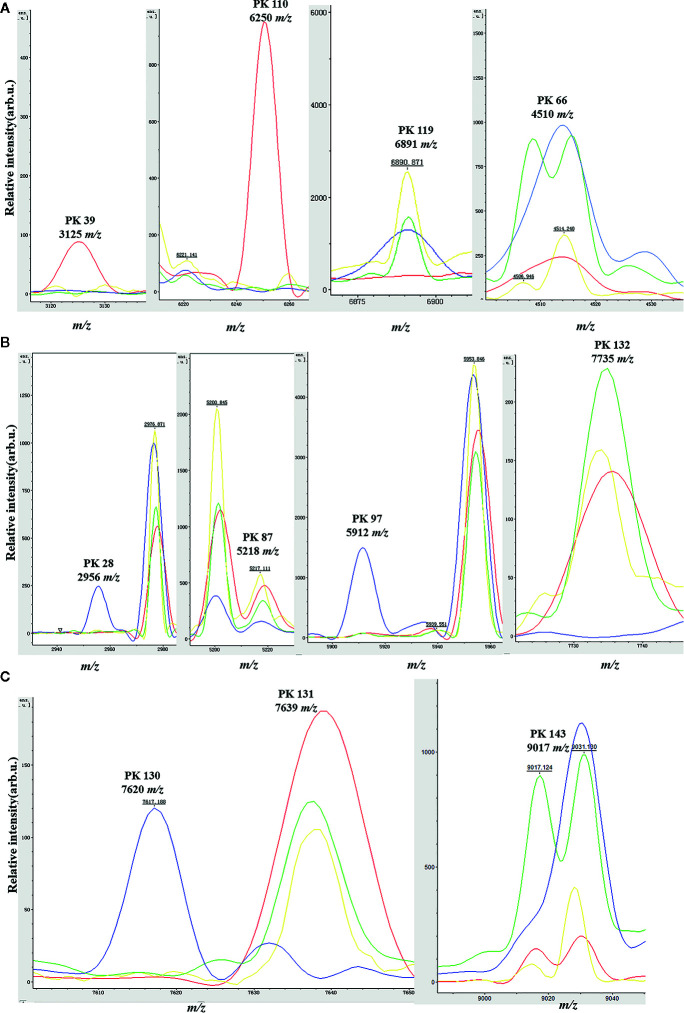
Spectra plots showing the presence or absence of the relevant peak biomarkers for MALDI-TOF/MS discrimination of the four main GBS clonal complexes in the genetic algorithm model. ST10 (red), ST12 (green), ST17 (blue); ST19 (yellow). x-axis shows the mass per charge ratio values (m/z) and y-axis indicates the intensities of peaks expressed in arbitrary intensity units. **(A)** Peak biomarkers for ST10 (m/z 3125, 6250, 6891) and ST12 (m/z 4510); **(B)** Peak biomarkers for ST17 (m/z 2956, 5218, 5912, 7735); **(C)** Peak biomarkers for ST10 and ST12 (m/z 9017), ST17 (m/z 7620).

**Figure 3:**
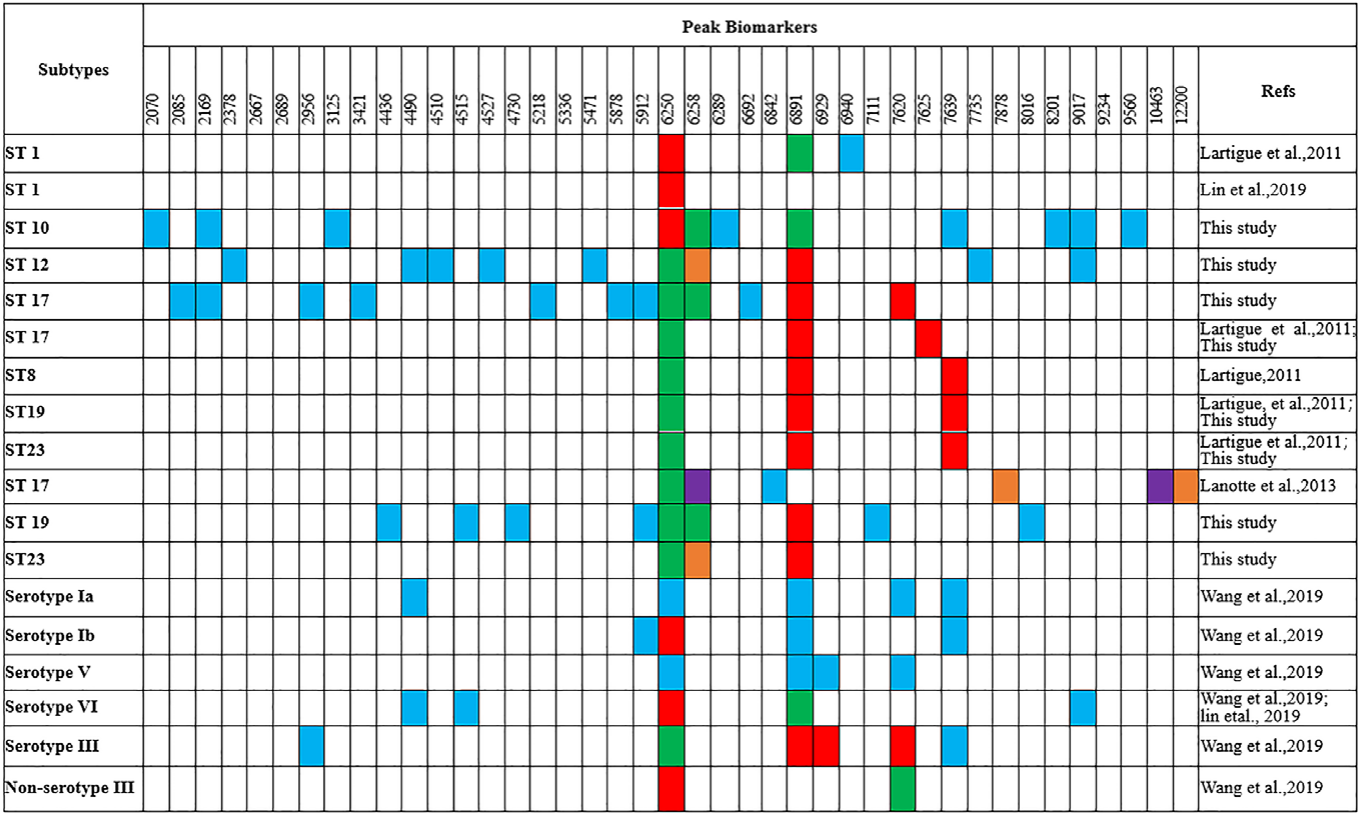
Comparison of the peak biomarkers used to detect ST1, ST10, ST12, ST17, ST19 of GBS strains by MALDI-TOF MS (m/z ±10) ^a^Red, specific presence of the peak; green, specific absence of the peak; blue, peak included in the typing model; orange, overexpressed peak; purple, under-expressed peak.

Peaks were identified by ClinProTools 3.0 software through GA-KNN algorithms. The most informative peaks for each predictive model were listed in **Table 2**. For all informative peaks, fourteen of them (m/z 2956, 3125, 4489, 4510, 4515, 4527, 5218, 5878, 5912, 6250, 6892, 6940,7735 and 9017) were statistically differential among four major STs (ST10, ST12, ST17, ST19) (**Table 3**). Most of the informative peaks from ST-GA(10)-KNN3 models were depicted with the peak intensity and frequency for six different subclasses (ST10, ST12, ST17, ST19, ST23, other STs) by ST subtyping MSPs peak data (**Table 4**). Three peaks (m/z 6250, 6891, 3125) and two peaks (m/z 2956, 5912) were characteristic informative peaks for ST10 and ST17 strains respectively, all displaying a statistical discriminative weight over 1.2. High intensity of peptide ions m/z 6891 (6888–6895) was found to be deficient in all ST10 strains instead of other STs. This study discovered an obvious peptide peaks at both m/z 2956 and m/z 5912 with a statistical weight of 1.42 and 1.83 respectively as top two powerful discriminative peaks for ST17 strains (**Table 2**). Both statistical (**Table 3**) and ST subtyping MSPs peak analysis (**Table 4**) supported the major discriminative peaks for ST10 (m/z 6250, 6891, 3125, 7639), ST17 (m/z 5912, 2956, 7735, 5218, 7620, 5878), ST12/ST19 (m/z 2956, 5912, 6250, 6891). ST subtyping MSP peak data (**Table 4**) also showed a low intensity of peptide ions at m/z 6261 other than m/z 6250 in both ST651 strains, 65.6% of ST12 strains and all ST23 strains. Specially, informative peaks from GA-KNN models showed a peptide ions peak at m/z 7620 as the discriminative peak for ST17, and m/z 7639 for ST10 (**Table 2**). MSP analysis displayed ST10 (67.7%), ST23 (45.4%), and ST19 (57.9%) had low average intensity of peptide ions at m/z 7639, ST12 (87.5%), and other minor STs (other STs, 71.8%) showed low intensity of peptide ions at m/z 7644, while ST17 (67.9%) strains displayed low average intensity of peptide ions m/z 7627 (**Table 4**). The peak shift (Sauget et al., 2017) from m/z 7639–7644 to m/z 7620, similar to the previous report (Lartigue et al., 2011), could differentiating III/ST17 strains from most other STs (mainly Ib/ST10, Ib/ST12, III/ST19, and other rare STs) with 48.24% sensitivity and 93.1% specificity. Few III/ST17 3.53% (3/85) strains also showed peptide ions at m/z 7630–7640. Mass spectra visualization for different STs supported the existing of characteristic peak markers for major STs, including the presence of m/z 6250 and 3125 and absence of m/z 6891 (6888–6895) for ST10 (serotype Ib) strains, as well as absence of peptide ions m/z 6250 along with presence of m/z 6891 (6888–6895) in nearly all other major STs (ST12/Ib, ST17/III, ST19/III, ST19/V, ST23/Ia), minor STs and even sporadic STs, and higher peaks at m/z 2956 and 5912 and lower peaks at m/z 7735 and 5218 in accompany with a peak shift from m/z 7644 to 7620 for ST17 strains similar to the previous report (Lartigue et al., 2011), as well as high peak at 6891 m/z, low peak at both 2956 m/z and 5912 m/z, no peak at both 7620 m/z and 6250 m/z for both ST12 and ST19 strains (**Table 2**, **Table 3**, **Table 4**, **Figure 2**). The pattern of these peaks for each STs and serotypes was shown in **Figure 3**, including the discriminative peaks by ST10, ST12, ST17, ST19 models in this study and the previous reported peak biomarkers for ST1 (Lartigue et al., 2011; Lin et al., 2019), ST17 (Lartigue et al., 2011; Lanotte et al., 2013), or serotypes (Wang et al., 2019).

**Table 2:** The ten discriminative peaks calculated by optimized GA-KNN models.

Peak no.	ST10		ST12		ST17		ST19		ST12/ST19	
	m/z	Weight	m/z	Weight	m/z	Weight	m/z	Weight	m/z	Weight
**1**	6250	2.50	4510	0.79	5912	1.83	5471	0.66	5912	1.28
**2**	6891	1.73	2956	0.65	2956	1.42	6250	0.64	2956	1.21
**3**	3125	1.29	6250	0.63	7735	0.48	5912	0.57	6929	0.51
**4**	6289	1.20	4527	0.55	5218	0.46	4436	0.41	6250	0.49
**5**	9016	0.64	9560	0.48	7620	0.43	8066	0.28	7620	0.45
**6**	6940	0.61	2378	0.35	5878	0.41	9235	0.20	6891	0.43
**7**	8201	0.33	6891	0.25	6693	0.29	4515	0.20	2689	0.32
**8**	7639	0.21	5471	0.24	2085	0.24	4730	0.20	5336	0.18
**9**	2169	0.08	4490	0.23	3422	0.21	6842	0.18	2667	0.17
**10**	2070	0.08	9017	0.19	2169	0.18	7111	0.04	3498	0.14

**Table 3:** Peak statistics among four major STs(ST10, ST12, ST17, ST19) displayed the 16 significant differential informative peaks from GA-KNN models.

Peak no^a^	Mass	DAve	PTTA	PWKW	PAD	Ave			
						ST10	ST12	ST17	ST19
28	2956	5.35	<0.001	<0.001	<0.001	1.08	1.21	6.43	1.41
39	3125	12.3	<0.001	<0.001	<0.001	13.04	0.95	0.7	0.88
65	4489	2	<0.001	<0.001	0.138	3.75	4.97	5.52	3.52
66	4510	6.51	<0.001	<0.001	<0.001	11.81	13.2	10.1	6.71
67	4515	6.51	0.011	0.026	0.003	11.76	16.3	17.5	18.3
68	4527	1.18	0.026	0.027	0.196	3.23	4.4	4	3.67
87	5218	3.75	0.005	0.017	<0.001	8.57	10.6	7.88	11.6
95	5878	0.72	0.005	0.002	<0.001	0.78	1.23	1.5	1.21
97	5912	16.9	<0.001	<0.001	<0.001	2.22	2.94	19.2	2.97
110	6250	39.3	<0.001	<0.001	<0.001	40.3	1.28	1.01	1.05
119	6891	26.3	<0.001	<0.001	0.073	7.12	30.5	33.4	32.4
121	6940	84	0.013	0.006	<0.001	146.9	62.9	73.8	92.6
130	7620	0.6	0.123	0.0603	<0.001	0.46	0.56	1.06	0.48
131	7639	2.16	0.405	0.907	<0.001	3.33	1.17	1.31	1.8
132	7735	1.78	<0.001	<0.001	<0.001	2.29	2.28	0.51	2.29
143	9017	3.09	0.138	0.002	<0.001	6.26	5.91	4.63	3.17

mass, m/z value; weight, relative contribution of each peak to the model; Ave, difference between the maximal and the minimal average peak area/intensity of all classes; PTTA, p value of t-/analysis of variance test; PWKW, p value of Wilcoxon/Kruskal–Wallis test (preferable for non-normally distributed data); PAD, p value of Anderson–Darling test, which gives information about normal distribution (p-value AD ≤ 0.05, non-normally distributed; p-value AD > 0.05, normally distributed); Ave, peak area/intensity average of each class.

^a^Peak number: correlative numbering of the peak in the average spectra.

**Table 4:** ST subtyping MSPs peak data showed the average intensity and frequency of the informative peaks (m/z ±10) from GA-KNN models in different ST classes.

Peak (m/z)****	Intensity (frequency, %)****						
	ST10	ST12	ST17	ST19	ST23	ST others	
3125	5.38(96.8)	–	–	–	1.01(54.5)		–
3422	15.51(100)	28.16(78.1)	32.7(96.4)	17.16(89.5)	26.64(100)		26.48(94.9)
4490	–	–	1.61(71.1)	–	–		–
4510^a^	11.34(96.8)	22.8(100) ^a1^	–	13.04(100)^a2^	9.65(100)		–
4730	41.67(100)	61.61(100)	39.73(91.7)	46.25(100)	32.51(100)		51.91(100)
5471	15.34(100)	15.14(100)	28.33(100)	18.89(100)	12.18(100)		15.76(100)
5878	0.62(93.5)	1.67(100)	2.47(100)	0.92(86.8)	0.91 (100)		1.44(79.5)
5912	1.66(90.3)	3.63(87.5)	15.89(90.5)	2.40(97.40)	10.09(100)		9.54(84.6)
6250^b^	22.63(100)	1.30(65.6)^b1^	–	–	1.42(100)^b1^		- (15.38)^b2^
6289	1.09(80.6)	–	–	–	–		–
6842	30.55(96.8)	32.37(90.6)	40.72(78.6)	29.02(97.4)	34.62(90.9)		40.46(89.7)
6891^c^	–	29.16(100)	27.16(97.6)	23.24(100)	17.71(100)		19.12(97.4)^c^
6940	90.65(100)	74.25(100)	82.16(100)	87.25(100)	97.2(100)		83.08(76.9)
7620^d^	4.29(67.7)^d1^	2.00(87.5)^d2^	1.17(67.9)^d3^	1.53(57.9)^d1^	6.00(45.5)^d1^		1.22(71.8)^d2^
7735	5.38 (96.8)	–	–	–	–		–
8066	–	3.16(75)	–	1.23(63.2)	–		–
8201	20.55(100)	33.9(96.9)	27.51(100)	21.17(100)	14.7(100)		23.24(100)
9017^e^	8.91(100)^e^	–	10.97(100)^e^	10.56(100)^e^	4.17(100)^e^		9.09(100)^e^

a, b, c, d, e represented a differential average peak among different STs. a1, 4527 m/z in ST12; a2, 4515 m/z in ST19; b1, 6261 m/z in ST12 and ST23; b2, the frequency of peak 6250 in other STs by visualization on FlexAnalysis; c, 6889 in other STs; d1, 7640 m/z in ST10, ST19 and ST23; d2,7644 m/z in ST12 and other STs; d3, 7627 in ST17; e, 9023-9027 in ST10, ST17, ST19, ST23, and other STs.

### External Validation of the Calculated GA Models

Spectra grouping for model external validation was similar as model generation in **Table 1**. Validation analysis of the five GA-KNN models for the four major STs generated the sensitivity, specificity, accuracy, positive predictive values, and negative predictive values (**Table 5**). GA-KNN models for ST10, ST17, and ST12/ST19 exhibited good performance with high sensitivity (95%–100%), specificity (91.46%–99.23%), accuracy (92.79%–99.29%), PPV (80%–92.68%), and NPV (94.32%–99.23%), indicating their excellent discrimination ability of ST10, ST17, ST12/ST19 subtypes from other major STs. GA-KNN models for ST12 and ST19 were less powerful to discriminate ST12 and ST19 from other major STs, probably because of the great mass spectra similarity between ST12 and ST19 strains according to 2D peak statistic overview. Model validation (**Table S3**) displayed a high accuracy of the major STs by GA-KNN models for ST10 (99.29%, 140/141), ST17 (94.74%, 90/95), and ST12/ST19 (92.79%, 103/111), with only one strain (ST17) misjudged by ST10 model, five strains (2 ST17, 1 ST12, 2 ST19) by ST17 models, eight strains (1 ST12, 1 ST10, 4 ST17, 2 ST23) by ST12/ST19 models, while the mis-judgement rate was higher by GA-KNN models of ST12 (19.26%, 26/135) and ST19 (29.10%, 39/134).

**Table 5:** Performance evaluation of the five optimized GA-KNN predictive models.

GA model	Sn (%)	Sp (%)	ACC (%)	PPV (%)	NPV (%)
ST10	100 (11/11)	99.23 (129/130)	99.29 (140/141)	91.67 (11/12)	99.23 (129/130)
ST12	75.00 (9/12)	81.30 (100/123)	80.74 (109/135)	28.13 (9/32)	97.09 (100/103)
ST17	95 (38/40)	94.55 (52/55)	94.74 (90/95)	92.68 (38/41)	96.30 (52/54)
ST19	70.59 (12/17)	70.94 (83/117)	70.90 (95/134)	26.09 (12/46)	94.32 (83/88)
ST12 and ST19	96.55 (28/29)	91.46 (75/82)	92.79 (103/111)	80.00 (28/35)	98.68 (75/76)

GA, genetic algorithm; Sn, sensitivity; Sp, specificity; ACC, accuracy; PPV, positive prediction value; NPV, negative prediction value. S_n_ =TP/(TP + FN), S_p_ = TF/(TF + FP), Acc = (TF + TP)/ (T F+ TP + FP + FN), PPV = TP/ (TP + FP), NPV = TN/ (TN + FN). TP, true positives; TN, true negatives; FP, false positives; FN, false negatives.

The classification results of minor and sporadic STs by all ML models as well as the number of isolates misjudged by five models to be four major STs were listed in **Table S4**. The repeated positively misclassification rate of these rare STs by ML models for ST10 was 75% (6/8), ST12 (50%, 7/14), ST17 (84.21%, 16/19), ST19 (56%, 14/25), ST12/ST19 (61.11%, 11/18), indicating better classification reproductivity of ST10 and ST17 models than ST12, ST19, and ST12/ST19 models (**Table S4**).

## Discussion

No MALDI-TOF/MS based statistical classification methodology has been reported for rapid GBS ST typing and peak biomarkers discovery yet. In this study, we have better characterized the MALDI-TOF/MS mass spectra of four main neonatal GBS STs (ST10, ST12, ST17, ST19) in China for rapid ST classification and investigation of potential peak biomarkers by applying ML to analyze MALDI-TOF spectra with ClinProTools 3.0. Our study, using gel overview and peak statistic view of mass spectra for different GBS STs, supports the theory that variations among GBS isolates were mainly due to the different protein expression profiles between different STs subtypes (Lartigue et al., 2009). As we known, the closer the cross-validation value and the recognition ability to 1, the better prediction ability the model probably have. Previous study also has proved nearly all the models with the highest the cross-validation value displayed highest Acc value (Wang H. Y. et al., 2018), which was usually used as another supporting index to select optimal models. Accordingly, GA-KNN models of ST10, ST17, and ST12/ST19 were more reliable and robust than ST12 and ST19 models, with a high cross validation value (97.92%–98.72%, **Table S2**) and good diagnostic efficacy (sensitivity, 95%–100%; specificity, 91.46%–99.23%; AAC, 92.79%–99.29%; PPV, 80%–92.68%, **Table 5**). This was in accordance with the clear separation among ST10, ST17, and ST12/ST19 by 2D peak statistical analysis (**Figure 1B**). All validated models showed NPV from 94.32% to 99.23%, indicating a high rate of correct negative classification result for mass spectra with unknown ST subtypes. Although most of the ST informative peaks in this study have been found to be discriminative peaks for different GBS serotypes, but no peaks have attained a discriminative score higher than 1.0 (Wang et al., 2019), probably because of enrollment of mass spectra with similar peak characteristics into different ML serotyping models, such as assignment of quantities of GBS spectra belonging to the same ST type (ST1, n = 120) but six different serotypes (Ia, Ib, II, III, V, VI) into different serotyping ML models.

For informative peaks, presence of high intensity of peptide ions m/z 6250 and absence of m/z 6891 (6888–6895) were found to be specific to ST10 similar to ST1 strains (Lartigue et al., 2011), but they were not specific biomarkers for serotypes Ia, Ib, III, V in this study or previous reported VI, III, Ib GBS isolates (Lin et al., 2019; Wang et al., 2019). Peptide ions of m/z 6250 were also found in sporadic STs [ST2(2/2, V), ST4(1/1, Ia), ST156(1/1, Ib), ST357(1/1, Ib), ST938(1, V)], they were repeatedly misjudged to be ST10 by ST10 ML model. Of note, ST4 was grouped as CC10/CC12 in along with ST10 (Ji et al., 2019). ST938 was classified as CC1 in along with ST1 (serotype V) which has specific peptide ions m/z 6250/6258 and a deficiency of peptide ions m/z 6888 (Lartigue et al., 2011). ST2 and ST156 were highly homogeneous to ST938, they were grouped as CC1 according to their genetic relationship in MLST clustering map of 31 ST subtypes (**Figure S1**). A protein peak of m/z 6251 (CsbD family protein) was detected in most (20/24, 92%) serotypes VI (94% ST-1 or single locus variant of ST-1), and a protein peak of m/z 6891 (UPF0337) protein gbs0600 was appeared in 83% serotypes III and Ib strains (Lin et al., 2019). The previous misunderstanding of m/z 6250/6251 presence and m/z 6891 absence with non-serotype III was probably due to the high association of serotype VI with ST1 (Lin et al., 2019), Ib with ST10, and III with ST17. The m/z 6891 protein in serotype III strains (Csb D-like protein) was supposed to be modified from m/z 6251 protein in serotype VI strains through the phage infection process with only seven amino acid residues differential to m/z 6251 protein (Lin et al., 2019).

This study also discovered obvious peptide peaks at both m/z 2956 and m/z 5912, with a statistical weight of 1.42 and 1.83 respectively, as the two most discriminative peaks for ST17 strains. MSP ST subtyping analysis showed the intensity of m/z 5912 was higher in ST17 than most other STs, but it was relatively high in ST23 and some other rare STs. Accordingly, we found the two ST17 strains that were misclassified to be non-ST17 during model external validation was mainly because of their low peak intensity at m/z 2956 and 5912, this misclassification could be corrected by simply replacement with high quality mass spectra. Moreover, some other STs including ST23 and ST188 that were repeatedly misclassified as ST17 also showed high intensity at peak m/z 2956 and m/z 5912. These STs were either classified to be CC17 (ST188, ST146) as ST17, or CC23 (ST24, ST223, ST249) as ST23 (Ji et al., 2019), or genetic highly homogeneous to ST23 (ST55), ST24 (ST163, ST452), ST188 (ST179), ST17 (ST680, ST146), indicating similar protein profiles could be detected between STs with highly homogeneous genetic relationships. The higher peak at m/z 5912 probably was a new biomarker for discrimination of ST17/III subtypes from other major STs. Interestingly, m/z 5912 is just two times of m/z 2956, indicating the peak at m/z 2956 is probably the doubly charged ion of m/z 5912, and hence they are not independent biomarkers. Previous study has identified a peptide peak at m/z 5911(which only exist in 15% non-Ib strains) to be a discriminative peak (rank 9) between Ib and non-Ib strains, while a peptide peak at m/z 2957 (rank 4, exist in 30% III and 4% non-III strains) between III and non-III strains (Wang et al., 2019). These were consistent with our result that high intensity at peak m/z 2956 and m/z 5912 was more specific to ST17 in comparison to non-ST17 strains (mainly ST10, ST12, and ST19), which has been reported to be informative peaks for serotype III and Ib recognition model respectively (Wang et al., 2019). Since the III/ST17 strains were mainly isolated from blood and CSF in neonates with invasive GBS infections, this newly identified peak biomarker m/z 5912 was indicative to the high pathogenicity of this clone and needed to be further illuminated. Consistent with the previous report (Wang et al., 2019), a peak shift from m/z 7644 to m/z 7620 (Lartigue et al., 2011) was also found for differentiating III/ST17 subtypes from other STs (Ib/ST10, Ib/ST12, III/ST19, etc). However, in this study, the peak shift from 7644 to 7620 could only differentiating III/ST17 strains from most other STs (mainly Ib/ST10, Ib/ST12, III/ST19, and other rare STs) with 48.24% sensitivity and 93.1% specificity compared to previous reported 100% specificity and sensitivity (Lartigue et al., 2011). This relative inconsistency as well as no statistical differences of m/z 7620 and 7639 among four STs (ST10, ST12, ST17, ST19) was probably because of peaks with very low intensity (below 500–1000 u.a) may remain undetected under certain acquisition conditions, with greater variations of frequency. Similarly, for other informative peaks like peak 132 (m/z 7735) and 143 (m/z 9017), although both peak statistical data and manual visualization supported they were differential peaks among four major STs (ST10, ST12, ST17, ST19), but MSP peak data did not show consistent frequencies and intensities in some STs. Noticeably, m/z 7620 showed similar mass weight and characteristic to p7878, a small subunit of exodeoxyribonuclease VII as catalyzer of exonucleolytic cleavage to maintain genome stability during DNA mismatch repair (Lanotte et al., 2013). p7878 was also overexpressed mainly in 97.83% ST17 clones (more than 4-fold more abundant in ST17 than other isolates), therefore, it was also of special interest as m/z 7620 as a highly discriminative peak for ST17 isolates from meningitis patients (Lanotte et al., 2013).

In summary, we have developed an accurate, fast and cost effective ML method in analyzing MALDI-TOF spectra of GBS strains for prevalent STs classification with 70.90% to 99.23% accuracies. Model external validation reveals GBS mass spectrum protein profiles are well characterized by clonal complexes (CCs), mainly including CC10, CC12, CC17, CC19, CC23 with differential invasive potentials and pathogenicity, indicating differential CC-dependent protein fingerprints in accordance with newly genomic finding of CCs lineage-specific genes associated with virulence, disease, metabolism, and regulation of cellular mechanisms (Gori et al., 2020). With ST dependent characteristic mass spectra profiles, especially for ST10 and ST17, we propose several possible applications for this method: (i) fast identification of human prevalent GBS strains like hypervirulent ST17 and multi-resistant ST19 clones both in neonates, pregnant women, or women with threatened preterm delivery and unknown colonization status; (ii) identification of new peak markers for different STs, which may promote the illumination of differential pathogenicity among different CCs of GBS strains. Our study also offers a new way for timely tracking the prevalent ST drifting of GBS population in large scale with the introduction of intrapartum antimicrobial prophylaxis (IAP). Limitations of this study include mis-judgement of GBS STs caused by mass spectra variations, limited discovery of ST specific peak markers and diagnostic efficiency for different STs as for limited STs subtypes, limited sample size of different STs in neonates, using ST subtyping but not CC typing during model generation, as well as dependence on special commercial statistic tool. Mass spectra for ML prediction were acquired only from in-tube extracted profiling instead of the most popular direct bacteria deposit for bacteria identification as previous report (Wang H. Y. et al., 2018), which may limit its clinical application. Variations that may related with the batch effect on ML prediction were still observed among replicates of mass spectra from the same isolates and STs. Application of averaged spectra from multiple MALDI-TOF measurements of the same isolates has been proved to be helpful to improve the diagnostic efficiency (Wolters et al., 2011; Camoez et al., 2016). Our pretest also supports this improvement could be achieved especially for ML models with relative low performance, such as GA-KNN models for ST12 or ST19, batch effect on ML prediction could be decreased either. Therefore, the future advance works will include: (i) increased collection of replicates of high qualified mass spectrum for each strain; (ii) application of CC typing instead of ST typing; (3) enroll GBS isolates from populations like pregnant and non-pregnant women; (iiii) for epidemical molecular monitoring of GBS diseases, development of automated ST classification methodology without commercial charged software like CliniProTools and diagnostic power evaluation of classification models for mass spectrum collected by both direct bacteria deposit as well as in-tube extracted protein is necessary in the future.

## Data Availability Statement

The original contributions presented in the study are included in the article/**Supplementary Material**. Further inquiries can be directed to the corresponding authors.

## Author Contributions

Conceptualization: HL, WJ. Methodology: LH, KG, GC, XG, QD. Formal analysis: LH, GC, ZL. Investigation: LH, GC, HZ, YX, C-YC. Writing—original draft: LH. Writing—review and editing: DM, WJ. Visualization: LH, HL. Funding acquisition: HL. All authors contributed to the article and approved the submitted version.

## Funding

This work was supported by grants from Guangzhou Science Technology and Innovation Commission (201804010447) and Guangzhou Women and Children Medical Center (YIP-2019-051).

## Conflict of Interest

The authors declare that the research was conducted in the absence of any commercial or financial relationships that could be construed as a potential conflict of interest.
