# Supplementary material for: Rapid Classification of Multilocus Sequence Subtype for Group B Streptococcus Based on MALDI-TOF Mass Spectrometry and Statistical Models
Source: Front Cell Infect Microbiol. 2021 Jan 29;10:577031. doi: 10.3389/fcimb.2020.577031 (PMC7878539; doi:10.3389/fcimb.2020.577031)
Supplement: Supplementary file 1 [file DataSheet_1.zip › Supplementary Table 1.DOCX]

**Table S1. The distribution of MLST types among various serotypes of GBS.** MLST, multilocus sequence typing. GBS, group B *Streptococcus*.

| **MLST** | **Serotype** | | | | | | |
| --- | --- | --- | --- | --- | --- | --- | --- |
|  | **Ia** | **Ib** | **III** | **Ⅳ** | **V** | **VI** | **Total** |
| 2 |  |  |  |  | 1 | 1 | 2 |
| 4 | 1 |  |  |  |  |  | 1 |
| 8 |  | 1 |  |  |  |  | 1 |
| 10 | 1 | 30 |  |  |  |  | 31 |
| 12 |  | 31 | 1 |  |  |  | 32 |
| 17 |  |  | 85 |  |  |  | 85 |
| 19 |  | 1 | 34 |  | 2 |  | 37 |
| 23 | 11 |  |  |  |  |  | 11 |
| 24 | 1 |  | 1 |  |  |  | 2 |
| 27 |  | 1 | 4 |  | 1 |  | 6 |
| 55 | 1 |  |  |  |  |  | 1 |
| 88 | 1 |  |  |  |  |  | 1 |
| 103 | 1 |  |  |  |  |  | 1 |
| 138 |  |  | 1 |  |  |  | 1 |
| 146 |  |  | 1 |  |  |  | 1 |
| 156 |  | 1 |  |  |  |  | 1 |
| 163 |  |  |  |  | 1 |  | 1 |
| 179 |  |  | 1 |  |  |  | 1 |
| 188 |  |  | 5 |  |  |  | 5 |
| 197 |  |  | 2 |  |  |  | 2 |
| 223 | 1 |  |  |  |  |  | 1 |
| 249 | 1 |  |  |  |  |  | 1 |
| 268 |  | 1 |  |  |  |  | 1 |
| 335 |  |  | 1 |  |  |  | 1 |
| 357 |  | 1 |  |  |  |  | 1 |
| 452 |  |  |  | 1 |  |  | 1 |
| 480 |  |  | 1 |  |  |  | 1 |
| 579 |  | 1 |  |  |  |  | 1 |
| 651 |  |  | 2 |  |  |  | 2 |
| 680 |  |  | 1 |  |  |  | 1 |
| 938 |  |  |  |  |  | 1 | 1 |
| Total | 19 | 68 | 140 | 1 | 5 | 2 | 235 |
