# Supplementary material for: Rapid Classification of Multilocus Sequence Subtype for Group B Streptococcus Based on MALDI-TOF Mass Spectrometry and Statistical Models
Source: Front Cell Infect Microbiol. 2021 Jan 29;10:577031. doi: 10.3389/fcimb.2020.577031 (PMC7878539; doi:10.3389/fcimb.2020.577031)
Supplement: Supplementary file 1 [file DataSheet_1.zip › Supplementary Table 2.DOCX]

**Table S2 Test performance of various lineage typing ML models**

| **ST** | **Model** | **Selected peaks** | **Cross validation(%)** | **Recognition (%)** |
| --- | --- | --- | --- | --- |
| ST10 | QC | 1 | 84.36 | 86.62 |
|  | SNN | 1 | 91.80 | 100.00 |
|  | GA(10)-KNN1 | 10 | 98.01 | 100.00 |
|  | GA(10)-KNN3 | 10 | 98.72 | 100.00 |
|  | GA(10)-KNN5 | 7 | 98.72 | 100.00 |
|  | GA(10)-KNN7 | 5 | 95.45 | 97.37 |
|  | GA(20)-KNN1 | 20 | 92.76 | 100.00 |
|  | GA(20)-KNN3 | 20 | 94.04 | 100.00 |
|  | GA(20)-KNN5 | 8 | 97.44 | 100.00 |
|  | GA(20)-KNN7 | 6 | 98.72 | 95.94 |
|  | GA(30)-KNN1 | 30 | 90.20 | 100.00 |
|  | GA(30)-KNN3 | 24 | 96.73 | 100.00 |
|  | GA(30)-KNN5 | 7 | 97.44 | 100.00 |
|  | GA(30)-KNN7 | 5 | 98.72 | 100.00 |
| ST12 | QC | 1 | 61.77 | 69.17 |
|  | SNN | 1 | 70.89 | 98.33 |
|  | GA(10)-KNN1 | 10 | 77.45 | 100.00 |
|  | GA(10)-KNN3 | 10 | 81.19 | 100.00 |
|  | GA(10)-KNN5 | 10 | 78.27 | 100.00 |
|  | GA(10)-KNN7 | 8 | 84.57 | 96.67 |
|  | GA(20)-KNN1 | 20 | 74.63 | 100.00 |
|  | GA(20)-KNN3 | 20 | 77.09 | 98.33 |
|  | GA(20)-KNN5 | 14 | 72.89 | 96.67 |
|  | GA(20)-KNN7 | 10 | 76.17 | 93.33 |
|  | GA(30)-KNN1 | 30 | 75.81 | 100.00 |
|  | GA(30)-KNN3 | 27 | 77.45 | 96.67 |
|  | GA(30)-KNN5 | 15 | 67.76 | 91.67 |
|  | GA(30)-KNN7 | 13 | 76.99 | 85.83 |
| ST17 | QC | 16 | 72.95 | 86.88 |
|  | SNN | 5 | 90.74 | 98.89 |
|  | GA(10)-KNN1 | 10 | 98.88 | 100.00 |
|  | GA(10)-KNN3 | 10 | 98.37 | 100.00 |
|  | GA(10)-KNN5 | 7 | 98.26 | 100.00 |
|  | GA(10)-KNN7 | 5 | 96.24 | 100.00 |
|  | GA(20)-KNN1 | 20 | 97.36 | 100.00 |
|  | GA(20)-KNN3 | 19 | 96.02 | 100.00 |
|  | GA(20)-KNN5 | 9 | 100.00 | 100.00 |
|  | GA(20)-KNN7 | 6 | 100.00 | 98.89 |
|  | GA(30)-KNN1 | 30 | 91.02 | 100.00 |
|  | GA(30)-KNN3 | 26 | 95.74 | 98.91 |
|  | GA(30)-KNN5 | 10 | 95.23 | 100.00 |
|  | GA(30)-KNN7 | 6 | 98.26 | 98.89 |
| ST19 | QC | 24 | 62.08 | 73.57 |
|  | SNN | 21 | 60.42 | 74.29 |
|  | GA(10)-KNN1 | 10 | 72.92 | 100.00 |
|  | GA(10)-KNN3 | 10 | 75.42 | 98.33 |
|  | GA(10)-KNN5 | 10 | 72.92 | 98.33 |
|  | GA(10)-KNN7 | 9 | 81.25 | 98.33 |
|  | GA(20)-KNN1 | 20 | 70.00 | 100.00 |
|  | GA(20)-KNN3 | 20 | 67.08 | 96.67 |
|  | GA(20)-KNN5 | 15 | 75.83 | 96.67 |
|  | GA(20)-KNN7 | 12 | 80.00 | 95.00 |
|  | GA(30)-KNN1 | 30 | 72.92 | 100.00 |
|  | GA(30)-KNN3 | 29 | 70.42 | 98.33 |
|  | GA(30)-KNN5 | 15 | 72.08 | 91.19 |
|  | GA(30)-KNN7 | 13 | 71.25 | 89.52 |
| ST12/ST19 | QC | 17 | 66.12 | 72.90 |
|  | SNN | 24 | 91.20 | 98.89 |
|  | GA(10)-KNN1 | 10 | 96.20 | 100.00 |
|  | GA(10)-KNN3 | 10 | 96.35 | 100.00 |
|  | GA(10)-KNN5 | 9 | 93.75 | 100.00 |
|  | GA(10)-KNN7 | 7 | 94.12 | 98.86 |
|  | GA(20)-KNN1 | 20 | 89.27 | 100.00 |
|  | GA(20)-KNN3 | 20 | 93.75 | 100.00 |
|  | GA(20)-KNN5 | 11 | 90.53 | 98.86 |
|  | GA(20)-KNN7 | 11 | 94.12 | 98.86 |
|  | GA(30)-KNN1 | 30 | 87.46 | 100.00 |
|  | GA(30)-KNN3 | 27 | 91.72 | 100.00 |
|  | GA(30)-KNN5 | 13 | 93.75 | 100.00 |
|  | GA(30)-KNN7 | 13 | 88.75 | 97.73 |

Abbreviations: GA, Genetic Algorithm; KNN, K Nearest Neighbor; SNN, Supervised Neural Network; QC, Quick Classifier. Detail methods for model optimization with GA-KNN algorithm: when GA algorithm was used as a method to select the peak combinations, the maximum number of best peaks was evaluated as 10, 20 and 30 respectively, the maximum number of generations was set as 500 for the GA algorithm to run to be sure it wouldn’t not be reached as the stop criteria to halt calculation (Bruker Daltonik GmbH user manual, 2011), the numbers of the k-nearest neighbors (k-NN) evaluated were 1, 3, 5, 7 for each binary classes separation (Bruker Daltonik GmbH user manual, 2011) (Wang et al., 2018).
