# Supplementary material for: Rapid Classification of Multilocus Sequence Subtype for Group B Streptococcus Based on MALDI-TOF Mass Spectrometry and Statistical Models
Source: Front Cell Infect Microbiol. 2021 Jan 29;10:577031. doi: 10.3389/fcimb.2020.577031 (PMC7878539; doi:10.3389/fcimb.2020.577031)
Supplement: Supplementary file 1 [file DataSheet_1.zip › Supplementary Table 3.DOCX]

| **Train** | **Group 1** | **Correct rate(%)*** | **Group 2** | **Correct rate (%)*** |
| --- | --- | --- | --- | --- |
| ST10 | ST10 | 100 (11/11) | Non-ST10 | 99.23 (129/130) |
|  |  |  | ST12 | 22/22 |
|  |  |  | ST17 | 74/75 |
|  |  |  | ST19 | 27/27 |
|  |  |  | ST23 | 6/6 |
| ST12 | ST12 | 75 (9/12) | Non-ST12 | 81.30 (100/123) |
|  |  |  | ST10 | 17/21 |
|  |  |  | ST17 | 63/75 |
|  |  |  | ST19 | 20/27 |
| ST17 | ST17 | 95 (38/40) | Non-ST17 | 94.55 (52/55) |
|  |  |  | ST10 | 16/16 |
|  |  |  | ST12 | 16/17 |
|  |  |  | ST19 | 20/22 |
| ST19 | ST19 | 70.59 (12/17) | Non-ST19 | 70.49 (83/117) |
|  |  |  | ST10 | 17/21 |
|  |  |  | ST12 | 6/22 |
|  |  |  | ST17 | 60/17 |
| ST12/ST19 | ST12/ST19 | 96.55 (28/29) | Non-ST12/ST19 | 91.46 (75/82) |
|  |  |  | ST10 | 10/11 |
|  |  |  | ST17 | 61/65 |
|  |  |  | ST23 | 4/6 |

**Table S3. Validation** **evaluation of five ST GA-KNN models**

*Correct rate= number of right classified isolates / number of totally validated isolates×100%
