# Supplementary material for: Rapid Classification of Multilocus Sequence Subtype for Group B Streptococcus Based on MALDI-TOF Mass Spectrometry and Statistical Models
Source: Front Cell Infect Microbiol. 2021 Jan 29;10:577031. doi: 10.3389/fcimb.2020.577031 (PMC7878539; doi:10.3389/fcimb.2020.577031)
Supplement: Supplementary file 1 [file DataSheet_1.zip › Supplementary Table 4.DOCX]

**Table S4: Classification results of minor and sporadic STs**

| **MLST** | **ST23 (11)** | **Minor and sporadic STs** |
| --- | --- | --- |
| ST10 | / | N=8: ST2(2)*, ST4*, ST156*, ST357*, ST651(2), ST938* |
| ST12 | ST23(2)^1^* | n=12: ST8, ST27(2)^1^*, ST103*, ST138, ST188(2)^1*^, ST249*, ST268*, ST357, ST651(2)^1^* |
| ST17 | ST23(7)^6^* | N=12: ST24(2)^1^*, ST55*, ST146*, ST163*, ST179*, ST188(5)^5^*, ST223*, ST249*, ST452*, ST480, ST680* |
| ST19 | ST23(4)^2^* | N=21:ST8*, ST24(1), ST27(5)^3^*, ST55, ST103*, ST138*, ST179, ST188(2)^1^* ST197*, ST249*, ST268, ST335, ST357*, ST480*, ST579, ST938* |
| ST12/ST19 | / | N=18: ST8*, ST24(1), ST27(5)^4^*, ST103*, ST138*, ST197(2)^1^*, ST249, ST268, ST335*, ST357*, ST579, ST651(2)^1^* |

*the ST subtype with duplicate qualified mass spectra[*log(S)*≥2.3] positively misclassified as ST10, ST12, ST17, ST19, ST12/19 during classification analysis. The number on the right upside of the ST subtype represented the number of isolates of this ST with duplicate positive misclassification.
