# Supplementary figures and images for: Rapid Classification of Multilocus Sequence Subtype for Group B Streptococcus Based on MALDI-TOF Mass Spectrometry and Statistical Models
Source: Front Cell Infect Microbiol. 2021 Jan 29;10:577031. doi: 10.3389/fcimb.2020.577031 (PMC7878539; doi:10.3389/fcimb.2020.577031)

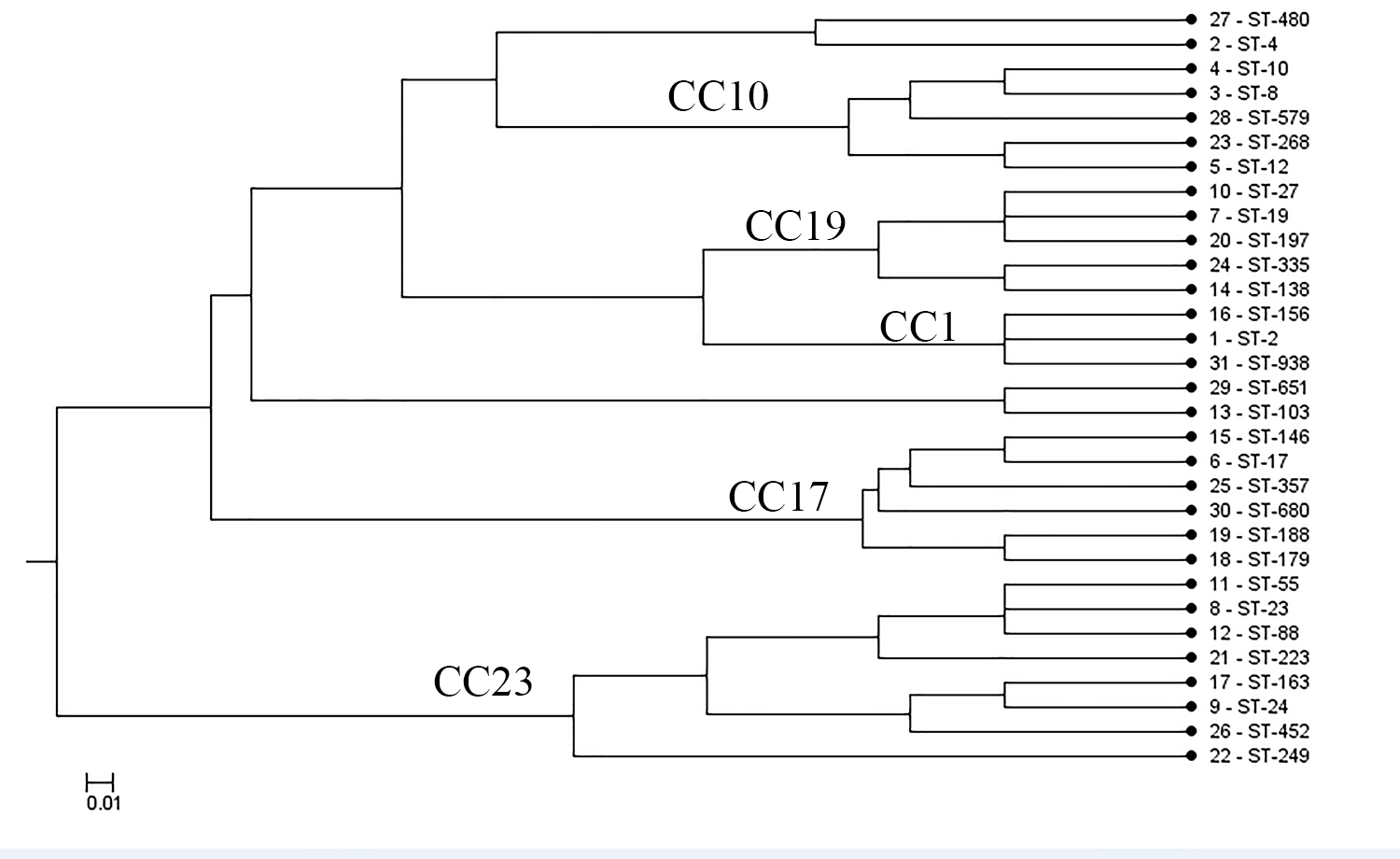

Supplement: Supplementary file 3 [file Image_1.tif]
